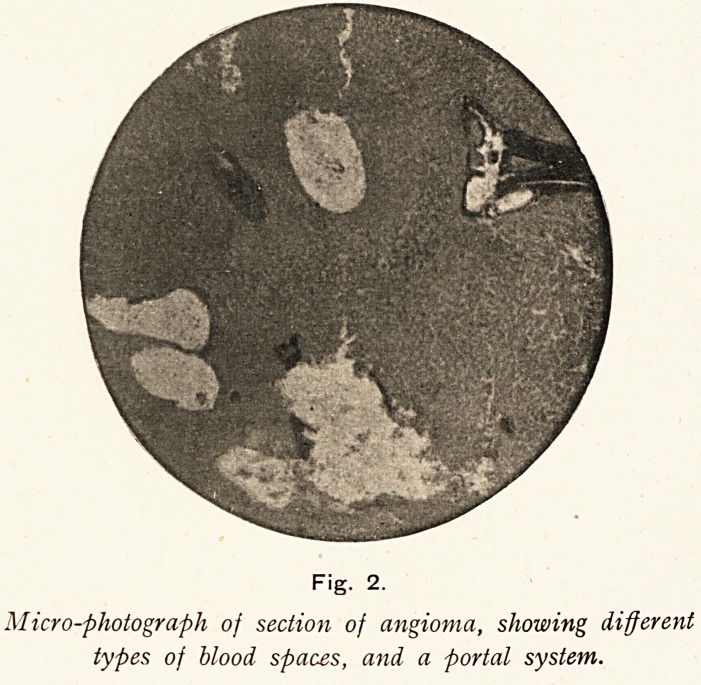# Acquired Angioma of the Liver

**Published:** 1907-03

**Authors:** A. Lewin Sheppard

**Affiliations:** House Physician to the Bristol Royal Infirmary.


### ACQUIRED ANGIOMA OF THE LIVER.

BY

A. Lewin Sheppard, M.B., B.S. Durh.,

*House Physician to the Bristol Royal Infirmary.*

---

The following article includes a description of a case recently in the wards of the Royal Infirmary under the care of Dr. Shaw, to whom I am indebted for permission to publish the case.

The patient, a man of 50, was admitted to the Bristol Royal Infirmary on November 21st, 1906.

*The history of his illness* was as follows :—About a year ago some " yellowness of the eyes " was noticed. Five months ago he began to complain of pain over the lower part of the chest and epigastrium. Three weeks ago he became worse while at work, and had to sit down, eventually going home to bed. After a fortnight in bed, during which the jaundice became more marked, he tried to put his clothes on, but found his trousers would not meet by several inches, owing to enlargement of the abdomen. He returned to bed, and the swelling increased somewhat rapidly until admission.

*Past history.*—An electrical engineer at a large factory for the last 25 years, he had lived in Bristol all his life with the exception of a few months at sea. He had enteric fever four years ago. No other illness. No hæmorrhoids. Rarely missed work. He drank a good deal of whisky. Appetite good, but occasional retching in the mornings. No specific history.

*Family history.*—Mother died of old age at 87. Father healthy, aged 89. His wife had had eleven children, of whom ten are alive ; eldest aged 23. No miscarriages.

*Condition on admission.*—Big, well-nourished man. Decidedly jaundiced. Temperature, normal ; pulse, 90 ; respirations, 28. Nothing abnormal found in heart or lungs. Arteries, not markedly thickened for his age ; slight œdema of legs. Urine (first 24 hours' specimen), 22 ounces, brown, s.g. 1012, cloud of albumen, bile. Abdomen, greatly distended ; signs of free fluid ; measurement, 43½ inches ; some large veins running upwards from lower part of abdomen ; enlarged liver just felt through fluid ; no pain.

*Progress.*—Abdomen increased in size and tension to 44 inches, when paracentesis abdominis was performed in the middle line through a $\frac{3}{16}$ inch trocar, the patient lying on his side.

Six pints, five ounces of semi-opaque fluid, typical of ascites due to portal obstruction ,was the result. The operation lasted 25 minutes, the fluid running slowly and the patient showing no signs of collapse. Immediately afterwards a nodular hard mass, the size of a tennis ball, was easily felt in the epigastrium, in consequence of which a malignant growth of the liver was suspected. The rest of the edge of the liver could not be made out. An abdominal binder was applied. This was the fifth day after admission. The following day he complained of pain in the abdomen, which now measured 42 inches, and during the following days the pain became more and more severe, the patient requiring both strychnine and morphia. Temperature, normal; pulse, feeble. This continued for nine days, when he died, the pain never having abated, and the fluid having rapidly reaccumulated.

*Post-mortem examination.*—Heart, normal. Vessels, healthy. Lungs, normal. Spleen, enlarged, hard and congested. Kidneys, enlarged and congested, bile stained. Pancreas, slightly harder than normal. Glands, no enlargement found, no malignant focus. No peritonitis. No angiomata found in other organs. Liver: weight 7 lb. 12 oz.; colour: brown, bile stained; capsule: adherent and of normal thickness; surface: masses of large, irregular nodules, most marked on the anterior surface, especially in the left lobe, which was enormously enlarged, and of a bluish colour, where it was soft and spongy. Antero-posterior section through the left lobe showed non-capsulated small dilatations throughout, varying in size from a pin's head to a pea; few in the posterior part, but closely packed together in the anterior segment. These dilatations contained blood, and the anterior part was discoloured and nearly black, fading posteriorly into the ordinary colour of an early cirrhotic liver. (*Vide* Fig. 1.)

*Microscopical sections* showed as the most marked feature a separation of the individual hepatic cells, *i.e.* intercellular spaces. In some collections of lobules this condition was exaggerated until cysts were formed, and large spaces were seen in various stages of development. In more recent ones were seen liver cells lying diffusely through the space, but the more cells so found the less distinct was the boundary of the cyst, formed as it was by the liver cells only. In the older spaces this condition was reversed, few or no cells lying within the space, but the edge was more defined, and in more marked cases the liver cells were lying concentrically, as if due to prolonged pressure. The portal systems on the whole were normal. (*Vide* Fig. 2.) In some places there was a slight appearance of cirrhosis and elastic tissue, while in isolated patches there is some small-celled infiltration around the blood sinuses throughout the lobules. The liver cells generally are neither atrophied nor damaged. Sections stained by Weigert's resorcin-fuchsin stain, in addition to the usual methods, demonstrated the absence of fibrous or elastic tissue in the small-celled infiltration which surrounded some of the larger cysts. The cystic dilatations were principally to be observed in the area of the hepatic veins, and appeared to develop from dilatation of the lobular veins, or by extravasation into the central areas of the lobules. These areas were previously in a state of advanced venous congestion, œdema being particularly well marked. The induration stage of passive hyperæmia was missing, and its absence may account for the unusual extent of the hemorrhages which are usually associated with that condition.

According to Rolleston,[1] angiomata of the liver can be injected from the hepatic artery, or from the hepatic or portal veins.

*Remarks.*—The case is of interest for several reasons.

Firstly, few are recorded. Lancereaux[2] mentions 25, and Schmieden[3] 32 cases, 18 of the latter being single and 14 multiple. They are said to be commoner in men, but Thoma[4] denies this.

Secondly, they may be acquired or congenital, and have been seen in fœtuses. (They are said to be comparatively common in cats.) According to Rolleston,[5] the acquired is the commoner variety, and they are then probably due to a combination of local congestion of the hepatic vessels and atrophy of the liver cells. Usually they are quite small, and only very occasionally have large cavernous tumours been seen in adults. In two such cases angiomata were found in other abdominal viscera. Large tumours of this type are usually encapsulated. The case in question was probably acquired, for the following reasons :—The age of the patient ; the definite history extending over a period of one year, with a more acute stage of about five months ; the irregular character of the condition as regards capsulation, and the extremely large size of the liver, which must have been noticed had it not increased very rapidly during the periods mentioned ; the complete absence of any past history of liver trouble ; and also, possibly, the slightly cirrhotic condition, connected as it was with a dis-

[1] *Encyclopædia Medica*, 1900, vi. 531.
[2] *Trait: des maladies du foie et du pancreas*, p. 528.
[3] *Arch. f. path. Anat.*, 1900, clxi. 373.
[4] *Pathology*, English translation by Bruce, i. 553.
[5] *Diseases of the Liver, Gall-bladder and Bile-ducts*, 1905, p. 461

Fig. 1.

*Photograph of antero-posterior section, showing darker area of angioma and spaces from which the blood has been washed out. Slightly less than half size.*

Fig. 2.

*Micro-photograph of section of angioma, showing different types of blood spaces, and a portal system.*

tinctly alcoholic history. The congenital condition is thought by some to be due to an excessive growth of the vascular mesoblast. The acquired type has been explained by a stagnation of blood, and congestion, which induce dilatation of the vessels with atrophy of the intervening liver cells. Some increased fibrosis takes place, so that a cavernous nævus is produced. This view is supported by Chervinsky,[1] and also by Hanot and Gilbert.[2]

The ascites, the pain, and the size of the tumour are all interesting features. The ascites possibly was accentuated by the cirrhosis also present, and the veins noticed on the abdominal wall would point to a portal obstruction that was not very recent. The pain and failure soon after paracentesis are difficult to explain, especially as the heart showed no signs of acute failure ; but the feeble nature of the pulse, and the rapid reaccumulation of fluid during the last few days, suggest a large amount of back pressure from the right side of the heart, probably causing a more acute dilatation of some of the more recently formed spaces. The size of the tumour is remarkable, especially for the unencapsulated form, in so old a subject. Mantle[3] mentions a case in a man aged 33, in which the liver weighed 6 lb. 13 oz., and the angioma hung down eight inches below the anterior edge of the liver, and was of the consistency of the placenta. The left lobe in this case was normal, except for some dilated branches of the portal vein. There was also small-celled infiltration and cirrhosis throughout, but there was no bile in the urine, and only a few ounces of fluid in the abdomen. The tumour was estimated to have contained eight pints of blood, and was only diagnosed after exploratory laparotomy, when a needle being put into the swelling blood spurted out, and, in spite of suturing and plugging, the patient died in two hours.

*The diagnosis* of such cases has usually only been made after laparotomy or death, but the venous hum sometimes heard over the liver area has been thought to be due to this cause. Hale

[1] *Arch. de Physiol. norm. et path.*, 1885, 3 s., vi. 553.

[2] *Études sur les maladies du foie*, 1888, p. 316.

[3] *Brit. M. J.*, 1903, i. 365.

White[9] says angioma is common, but produces no symptoms during life.

*Surgical treatment* by electrolysis is mentioned by Keen[1] as having been performed in four cases, and Cripps has recently removed a capsulated nævus of the liver, previously diagnosed as an ossifying sarcoma, with success. In 1894 Tenedat refers to only six cases in which excision has been carried out.

I am greatly indebted to Professor Walker Hall for laboratory assistance, and for the suggestion to investigate the subject.

The accompanying photograph I took to show the cyst-like spaces, &c., as explained above. For the micro-photograph of the section I am indebted to Mr. James Taylor.

[1] Allbutt's *System of Medicine*, 1897, iv. 211.
[2] *Ann. Surg.*, 1899, xxx. 276.

## Figures and Tables

**Fig. 1. f1:**
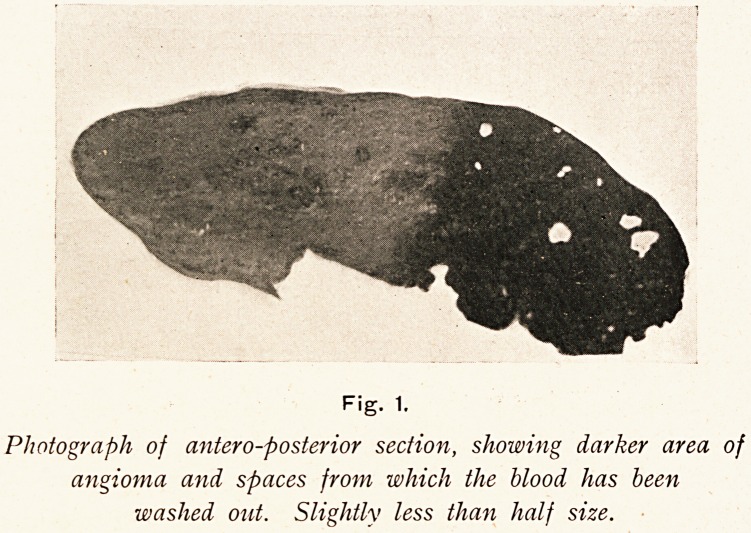


**Fig. 2. f2:**